# Shuffle & untangle: novel untangle methods for solving the tanglegram layout problem

**DOI:** 10.1093/bioadv/vbac014

**Published:** 2022-02-28

**Authors:** Nghia Nguyen, Kurdistan Chawshin, Carl Fredrik Berg, Damiano Varagnolo

**Affiliations:** 1 Department of Geoscience and Petroleum, NTNU, 7031 Trondheim, Norway; 2 Department of Engineering Cybernetics, NTNU, 7034 Trondheim, Norway; 3 Department of Information Engineering, University of Padova, 35122 Padova, Italy

## Abstract

**Motivation:**

A tanglegram is a plot of two-tree-like diagrams, one facing the other, and having their labels connected by inter-tree edges. These two trees, which could be both phylogenetic trees and dendrograms stemming from hierarchical clusterings, have thus identically labelled leaves but different topologies. As a result, the inter-tree edges of a tanglegram can be intricately tangled and difficult to be analysed and explained by human readers. To better visualize the tanglegram (and thus compare the two dendrograms) one may try to untangle it, i.e. search for that series of flippings of the various branches of the two trees that minimizes the number of crossings among the inter-tree edges. The untanglement problem has received significant interest in the past decade, and several techniques have been proposed to address it. These techniques are computationally efficient but tend to fail at finding the global optimum configuration generating the least tangly tanglegram.

**Results:**

We leverage the existing results to propose untanglement methods that are characterized by an overall slower convergence method than the ones in the literature, but that produce tanglegrams with lower entanglements.

**Availability and implementation:**

One of the algorithms is implemented in Python, and available from https://github.com/schlegelp/tanglegram.

## 1 Introduction

A common strategy to visually compare two different dendrograms relative to the same sets of terminal vertices is by using tanglegrams, i.e. comparative drawings (embeddings) of a pair of dendrograms, side-by-side, with matching objects connected by straight-line segments called inter-tree edges (Andreas *et al.*, 2012). A crossing occurs in a tanglegram when two inter-tree edges intersect, while there is no crossing inside the individual trees. The number of crossings, in its turn, depends on the layout (also known as drawing) of the two dendrograms in the tanglegram. In practical terms, layouts, which lead to a high number of crossings are harder to interpret (Andreas *et al.*, 2012). This calls for finding a drawing of the tanglegram with as few crossings as possible. Finding a tanglegram layout (TL) of two trees that produces zero crossings, sometimes referred to as a drawable layout (Fernau *et al.*, 2010), is known as the planar embedding problem (Andreas *et al.*, 2012). It is known that every pair of trees of size smaller or equal to three has a drawable layout (Fernau *et al.*, 2010). However, only special cases allow for a layout with zero crossings. The problem of finding a graphical layout of two trees that gives the minimum number of crossings is known as the TL problem (Bansal *et al.*, 2009), or the two-tree crossing minimization (TTCM) problem (Fernau *et al.*, 2010). The TTCM problem is known to be NP-complete, meaning that there is no known algorithm to solve this problem in polynomial time with the tree size, but only in exponential time (Fernau *et al.*, 2010).

When one tree is fixed, the problem above is referred to as the one-tree crossing minimization (OTCM) (Fernau *et al.*, 2010). For arbitrary trees, the OTCM problem can be solved in O(n log n) time (with *n* the number of leaves) as in Venkatachalam *et al.* (2009) and Fernau *et al.* (2010). Bansal *et al.* (2009), instead, presented an $O(n{\;\text{log}\;}^{2}n/\;\text{log}\;\;\text{log}\;n)$ algorithm for a generalized tanglegram.

A heuristics-based algorithm to tackle the TTCM problem was proposed in Dwyer and Schreiber (2004). Here, the authors repeatedly solve the OTCM problem for each tree, and eventually provide 2.5D graphical representations of the results. Another approximation algorithm for complete tanglegrams is provided in Buchin *et al.* (2009).

The TTCM for binary trees problem was shown in Fernau *et al.* (2010) to be fixed parameter tractable (FPT) with parameter *k* (the number of crossings). A number of FPT algorithms have then been introduced to address the TL problem. For example, an $O(1.4656^{k}+kn^{2})$ FPT algorithm for binary OTCM was introduced in Dujmović *et al.* (2008). We also note that the TTCM problem is solvable in $O(4^{k}n^{2})$ time if the optimal TL with *k* inter-tree edge crossings exists (Buchin *et al.*, 2009).

To the best of our knowledge, the latter and the algorithm in Dwyer and Schreiber (2004) were first compared against the *bb-1st-sol* algorithm (that has a running time of $O(n^{2})$) in Nöllenburg *et al.* (2009). Another fixed-parameter algorithm for TTCM problem with a complexity $O(c^{k}n^{O(1)})$, where *c* is estimated to be 1024, was proposed in Fernau *et al.* (2010). Besides, Scornavacca *et al.* (2011) introduced a heuristic approach that can solve the general case rooted phylogenetic networks and check if a tanglegram has a layout of zero crossings.

As shown above, there is a range of different methods for solving the TL problem. A suite of different algorithms are implemented in the dendextend package in the *R* language (Galili, 2015). Such package allows to visualize and compare trees of hierarchical clustering by offering a set of functions for extending dendrogram objects in *R*. More precisely, this package currently offers the following methods for solving the TL problem: *labels*, *ladderize*, *random*, *DendSer*, *step1side* and *step2side*. As shown below, among these methods *step2side* is the one that gives the lowest entanglement for our test cases, however at the highest computational cost. However, even if performing better in terms of returned entanglement levels, still *step2side* may fail in detangling pairs of trees that are detanglable. Our motivation is thus that of developing untangle methods that lower the entanglement even further than *step2side*.

In this article, we then introduce three new concepts: first, an extension of *step1side* and *step2side* that allows for a rotation in both dendograms simultaneously—a method that is denoted as *stepBothSides*. Then, due to the high computational cost of such *stepBothSides* method, we propose an additional strategy that shuffles the tanglegrams to obtain new starting positions for the *step2side* approach—a method that is denoted as *shufS2S*. Finally, we provide a final method that replaces the *step2side* method with a new untanglement strategy—denoted below as *Shuffle and Untangle*, and abbreviated with the name *ShUnTan* (this method is implemented here: https://github.com/schlegelp/tanglegram). This latter method aims at eliminating crossings that *step2side* fails to untangle (at the cost, however, of higher computational requirements than *step2side* for small tanglegrams).

The article is organized as follows: we first present fundamental notations and equations related to dendrograms and tanglegrams in Section 2. We then present the *step2side* algorithm in Section 3, and continue with introducing a new method that utilizes the result of *step2side* for further untangling in Section 4. In Section 5, we discuss the shuffling scheme and how it can improve the *step2side* method. We then introduce our proposed approach to sequentially shuffling the layout in Section 6. In Section 7, we deal with numerical examples that illustrate the performance of the various methods, and finally draw some concluding remarks in Section 8, where we also suggest some further research directions.

## 2 Preliminaries and notation

In this section, we will present the notation and background needed for defining the algorithms discussed and proposed in the article.

###  

####  

*Dendrograms and tanglegrams:* A tanglegram is the visualization of a pair of dendrograms side-by-side, with matching objects connected by straight-line segments called inter-tree edges (Andreas *et al.*, 2012). A dendrogram is a graphical visualization of a linkage matrix, which is a (n−1)×4 matrix, here denoted by *Z*, where the first two columns of *Z* store the indices of objects, which are combined to create a new group, the third column stores the distance between these objects and the fourth column stores how many of the *n* original objects end up being in the newly merged group. A toy example of a linkage matrix resulting from clustering a set of four objects (i.e. the indexes 1,2,3 and 4) is

(1) $$Z=\left[\begin{array}{cccc} 1 & 2 & 0.3 & 2\\ 3 & 5 & 0.2 & 3\\ 4 & 6 & 0.7 & 4 \end{array}\right].$$

Note that the distance between the objects will not be used in this article, as we are only interested in the topology of the dendograms.

A tanglegram is uniquely defined by a matrix $L=\left[Z_{l},Z_{r}\right]$, with *Z_l_* and *Z_r_* the linkage matrices of the left and right dendrograms. To such matrices correspond moreover the two sets *V_l_* and *V_r_*, denoting the *n*-dimensional vectors of ordered leaf nodes appearing at the ends of the left and right dendrograms.

**Fig. 1.**
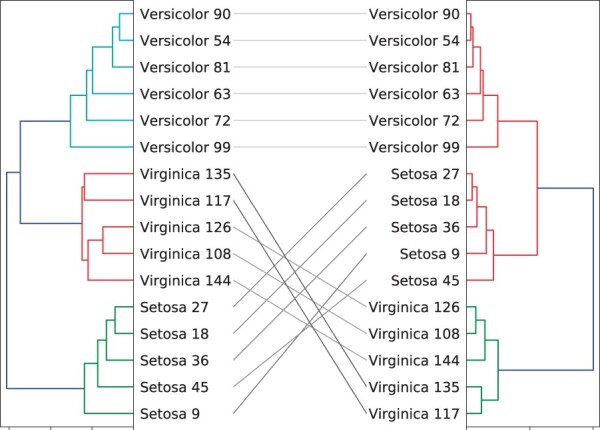
An example of a tanglegram obtained by performing a single linkage clustering (left dendrogram) and a complete linkage clustering (right dendrogram) on 16 samples of the Iris flower dataset (Fisher, 1936)

As an example, consider the 16 samples from the Iris flower dataset (Fisher, 1936) that appear as labels in Figure 1. The dendrograms on the left and right sides in Figure 1 correspond to clustering using a single linkage and a complete linkage clustering, respectively. The black lines in the middle represent the tanglegram connecting these two dendrograms. Each ‘branching’ in each dendrogram (and its height) reflects the values within the corresponding row in the linkage matrix. The two side-by-side dendrograms shall be intended as two distinct outputs of two different clustering algorithms on the same set of original objects.

Note that in the figures, the leaf nodes vectors *V_l_* and *V_r_* are represented with labels; actually this is a sort of abuse of notation, since formally they are ordered sets of Indices 1,2,…,n. The figures typically substitute then these indexes with the corresponding labels through an opportunely defined look up table (e.g. in Fig. 1, ‘Setosa 9’ may correspond to Index 1, ‘Setosa 45’ to Index 2, etc.).

####  

*Untangling a tanglegram through swapping operations*: Tanglegrams help comparing trees and dendrograms defined on the same sets of leaves, since they can highlight similarities and dissimilarities. However, to aid interpretability, one wishes to minimize the crossings among the various inter-tree edges in a tanglegram. To do so one may ‘swap’ two children clusters in an interior vertex in a dendrogram, an operation that from a graphical perspective corresponds to rotating a sub-tree in one of the dendrograms. Formally this means opportunely swapping the values of the first two columns in some of the rows of the linkage matrix. This function, denoted by $\Omega_{i}(Z)$, takes thus the linkage matrix *Z* as input, and returns an updated linkage matrix Z′ whose first two elements of the *i*th row of *Z* are swapped. Note that these rotation operations change the ordered leaf nodes vector *V*, but do not change the splittings (and thus the topology) of a dendrogram. In other words, applying a series of swaps $\Omega_{i}(Z)$’s does not alter the interpretability of the results of a clustering algorithm.

####  

*Displacement and entanglement*: Minimizing the number of crossings among the inter-tree edges in tanglegrams aids interpretability. Counting the number of crossings is a O(n log(n)) problem, where *n* is the tree size (Barth *et al.*, 2002). If this operation is repeatedly performed inside some untangling algorithms, then this could lead to a significant computational cost. Entanglement is an alternative measure that may be obtained with a lower computational cost and that may still indicate how tangled the two dendrograms are through an index that varies between 0 (drawings with no crossings) and 1 (a drawing with the worst layout possible) (Galili, 2015).

The definition of entanglement requires the definition of *displacement*; the displacement of $L=[Z_{l},Z_{r}]$ is calculated as the *p*-norm distance of *V_l_* and *V_r_* (Galili, 2015) (recall that the labels seen in the figures actually correspond to natural numbers, so that *V_l_* may be thought as the vector $V_{l}=[1,2,\ldots ,n]$ and *V_r_* as a shuffling of these indexes). For simplicity of treatment, though, in this study, we consider *p* = 2 (i.e. Euclidean norms).

The entanglement of *L*, indicated with ϵ(L), is then the ratio between its displacement, as defined above, over the worst displacement possible for tanglegrams of that size (i.e. the displacement that would be obtained when *V_r_* is the inverse of *V_l_*). This ratio guarantees that ϵ(L)∈[0,1]. As an example, the Iris example in Figure 1 has an entanglement of 0.207.

## 3 The *step2side* algorithm

As said above, untangling helps interpretability. However, untangling by hand is time consuming and prone to suboptimal results (as a riddle, the interested reader may try to untangle the tanglegram in Fig. 1). A series of algorithms that automate this task are implemented in the *R* package dendextend (see Galili, 2015), among with functions and tools for manipulating dendrograms structures. Among the six untangle methods implemented in this *R* package, to the best of our knowledge, the most effective and commonly used one is the so-called *step2side*.

For the pseudo-code of this algorithm and for subsequent developments, we will use a function $\xi \left(L_{1},\ldots ,L_{m}\right)$ that selects the tanglegram with the smallest entanglement among a set of *m* candidates. Formally,

(2) $$\xi :\{L_{1},\ldots ,L_{m}\}\mapsto \arg\underset{L}{\min}\epsilon (L).$$

The *step2side* method basically executes three main steps, starting from an original tanglegram $L_{0}=\left[Z_{l},Z_{r}\right]$:

Step 1: create new alternative TLs where the left dendrogram *Z_l_* is unchanged, while the right dendrogram layout *Z_r_* is altered by rotating one after the other the inner branches via the use of $\Omega_{i}(Z_{r})$, with 1≤i≤n−1. If $\epsilon ([Z_{l},\Omega_{i}(Z_{r})])<\epsilon ([Z_{l},Z_{r}])$, then *Z_r_* is replaced by $\Omega_{i}(Z_{r})$, thus $\left[Z_{l},Z_{r}]=\xi ([Z_{l},Z_{r}],[Z_{l},\Omega_{i}(Z_{r})]\right)$.
Step 2: similar to Step 1, but this time keeping the right dendrogram *Z_r_* unchanged and rotating the left one, and then compute the new entanglement level: $\left[Z_{l},Z_{r}]=\xi ([Z_{l},Z_{r}],[\Omega_{i}(Z_{l}),Z_{r}])\;\forall i\in [1,n-1\right]$.
Step 3: repeat Steps 1 and 2 until the entanglement does not reduce any further (i.e. the algorithm has found a local optimum).

**Fig. 2.**
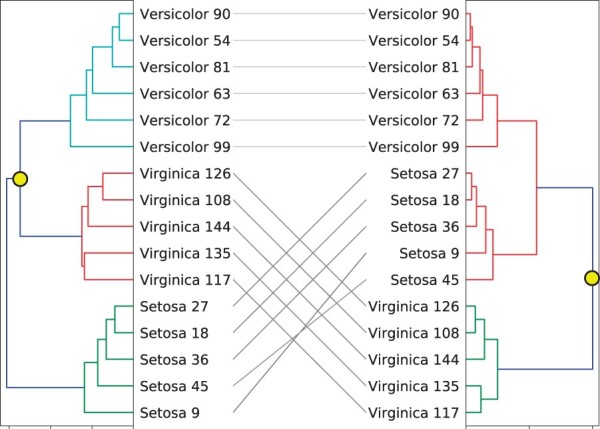
The resulting TL after using *step2side* on the layout shown in Figure 1. Applying the method lowers the entanglement to a value of 0.185. However, this may be lowered even further by simultaneously applying $\Omega_{14}(Z_{l})$ (i.e. swapping the ‘virginica’ and ‘versicolor’ blocks in the left dendrogram by rotating the second splitting, denoted by the left yellow dot) and $\Omega_{15}(Z_{r})$ (i.e. swapping also the ‘virginica’ and ‘versicolor+setosa’ blocks in the right dendrogram by rotating the first splitting, denoted by the right dot). This highlights that *step2side* suffers from stopping in local optima

As an example, applying *step2side* to the tanglegram in Figure 1 leads to the result in Figure 2. We can note that *step2side* reduces the entanglement value from 0.207 to 0.185, which could be considered marginal. It is also hard to distinguish the difference in complexity of the two tanglegrams from a visual inspection. Moreover, one can notice that *step2side* did not find the best layout possible, since the entanglement can be decreased further by simultaneously rotating clusters at the root of the left dendrogram and at the second last interior vertex of the right dendrogram, as also specified in the caption of the figure. This indicates that in general *step2side* reaches local minima, and thus there is room for potential improvements.

**Fig. 3.**
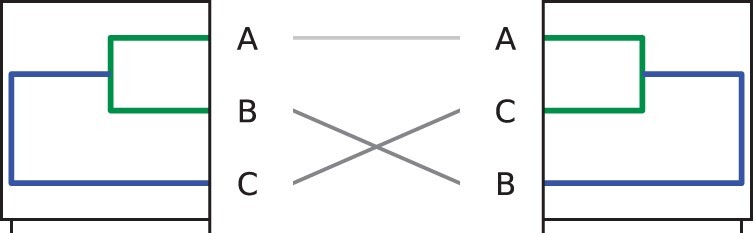
A simple tanglegram of three objects showing the limitation of *step2side* algorithm, that indeed fails in finding the 0 crossing TL given that the initial layout is not shuffled

The *step2side* algorithm presented above may stop in local optima: to prove this, consider Figure 3, with a tanglegram $L=\left[Z_{l},Z_{r}\right]$ consisting of three objects, *A*, *B* and *C* [thus, a tanglegram for which the optimal layout with zero crossing exists (Fernau *et al.*, 2010)]. Starting from the initial layout of Figure 3, the zero crossing layout can be found by first rotating the left tree at its root, and then rotating the green split in the right diagram. In other words, the optimum may be found first performing a ‘shuffling’ operation, and then applying the *step2side* algorithm. This realization leads to the consideration that it may be beneficial to carry on an additional ‘shuffle’ step, where two swaps are performed simultaneously. This consideration leads then to the first algorithm we propose in this manuscript, formalized in details in Section 5.

## 4 The *stepBothSides* algorithm

Recall the simple example in Figure 3, where the zero entanglement layout can be found by first swapping the left tree at the root and then applying *step2side*. Inspired by this improvement, below we extend *step2side* into a new algorithm that introduces an additional step rotating both sides simultaneously. The new algorithm thus still uses *step2side* to reduce the entanglement before applying the more computationally costly rotation at both sides. This algorithm will be referred to as *stepBothSides*, to highlight the similarity and distinctions from *step2side*.

**Fig. 4.**
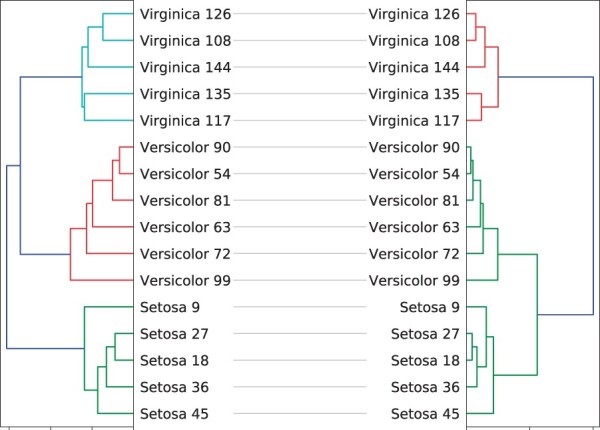
The TL after applying *stepBothSides* on the original layout in Figure 1. Contrary to *step2side*, *stepBothSides* succeeds in achieving zero entanglement

Consider receiving the same input tanglegram as *step2side*, i.e. $L_{0}=\left[Z_{l},Z_{r}\right]$. The *stepBothSides* algorithm performs then the following steps:Step 2, that represents an addition to the *step2side* algorithm, helps exiting the basin of attraction of the local optima encountered in the examples shown in Figures 2 and 3. Indeed, applying *stepBothSides* on the tanglegram in Figure 1 leads to the result shown in Figure 4—a tanglegram with zero crossings, thus, an entanglement value of 0.

Step 1: run the *step2side* algorithm until convergence;
Step 2: create new alternative tanglegrams $L_{ij}=[\Omega_{i}(Z_{l}),\Omega_{j}(Z_{r})]$ for all 1≤i≤n−1 and 1≤i≤n−1. Whenever $\epsilon (L_{ij})<\epsilon (L_{0})$, then replace *L*_0_ with *L_ij_*, i.e. $L_{0}=\xi (L,L_{ij})\forall i,j\in [1,n-1]$.
Step 3: repeat Steps 1 and 2 until the entanglement does not reduce any further.

This improvement comes though at a significant computational cost. Indeed, while the number of layouts to test in Step 1 is 2*n*, the number of test cases in Step 2 is *n*^2^. Step 1 is similar to the *step2side* algorithm and thus has a similar computation cost from an order of O(n) operations. Considering though Step 2 above, the overall number of test cases for *stepBothSides* is of the order $O(n^{2})$.

One may also generalize *stepBothSides* as follows: instead of rotating two, one may rotate four branches simultaneously. This would increase the computational cost even further, with the number of entanglement calculations of the order of $O(n^{4})$, but at the same time this would add flexibility in the search for the global optimum.

The generalization above may be pushed even further, with even more branches rotating simultaneously. This would though lead to a growth in the computational costs that may make this class of algorithms prohibitive for larger tree sizes. Inspired by these considerations, in the next sections, we thus introduce iterative algorithms that test deeper into the tree only when increasing such exploration depth shows potential for improvements.

## 5 The *shufS2S* algorithm

As mentioned at the end of Section 3, adding an additional ‘shuffle’ step to the *step2side* algorithm can aid reaching lower entanglement levels. In other words, inspired by the same considerations motivating the *stepBothSides*, the intuition is that allowing for swaps in both tanglegrams simultaneously may increase the chance of reaching better entanglement minima. Though, this shuffling means starting the untangling process from four possible TLs, increasing the computational cost. Moreover, shuffling for all possible internal connections in the two tanglegrams, would make the computational cost prohibitive. There is the need therefore for a heuristic that prevents excessive computational loads. Our proposal is then to start shuffling at the nodes near the roots of the two trees, and continue step-wise away from the root with an increasing number of shuffle operations if an improvement is observed, otherwise stop this process.

This intuition is the backbone of the algorithms detailed in the remainder of the article, where we introduce the ‘shuffle’ step above in two different ways, and describe how this type of step can be combined with *step2side* algorithm. We start with a first approach, referred as the *shuffle and step2side* algorithm (or *shufS2S* algorithm). To detail the method, we start by defining some ancillary functions invoked in the various individual steps of this algorithm.

### 5.1 Ancillary functions

Both the *shufS2S* and *ShUnTan* methods (the latter to be introduced in the next section) leverage the following set of functions:

1. The shuffle operation $\mu_{ij}(L)$, that takes as input a tanglegram *L* and returns four different tanglegrams, corresponding to rotating the left dendrogram at vertex *i* or not (i.e. applying or not $\Omega_{i}(Z)$ on the left) and rotating the right dendrogram at vertex *j* or not (i.e. applying or not $\Omega_{j}(Z)$ on the right dendrogram). Formally,
  (3) $$\mu_{ij}(L=[Z_{l},Z_{r}])=\left\{\begin{array}{c} L_{1}=L=[Z_{l},Z_{r}],\\ L_{2}=[\Omega_{i}(Z_{l}),Z_{r}],\\ L_{3}=[Z_{l},\Omega_{j}(Z_{r})],\\ L_{4}=[\Omega_{i}(Z_{l}),\Omega_{j}(Z_{r})] \end{array}\right\}.$$
  When *i* = j, we simply denote the function as *μ_i_*;
2. The function τ(i,Z), which returns the location of node *i* in the dendrogram *Z* (in other words, at which row of *Z* the object *i* appears). As an example, if *Z* is given by the matrix in (1), then τ(5,Z)=2. Formally,
  (4) τ(i,Z)=t where tis s.t.Z[t,1]=i or Z[t,2]=i.

### 5.2 The *shufS2S* in pseudo-code

Starting with the tanglegram $L_{0}=\left[Z_{l},Z_{r}\right]$, the *shufS2S* algorithm contains the following steps:

Step 1: initiate *m* = 0 and $L_{\text{ini}}^{\left(0\right)}=\{L_{0}\}$. The physical meaning of the parameter *m* will be further discussed in Subsection 6.2;
Step 2: increase *m* by 1 and shuffle all TL(s) in $L_{\text{ini}}^{\left(m-1\right)}$, i.e. $L_{i}\in L_{\text{ini}}^{\left(m-1\right)}$, at the ${\left(n-m\right)}^{th}$ interior vertex by using the swap function $\mu_{n-m}$
  (5) $$L_{\text{ini}}^{\left(m\right)}=\overset{k}{\underset{i=1}{\cup }}\mu_{n-m}(L_{i}),\;L_{i}\in L_{\text{ini}}^{\left(m-1\right)},$$
  where *k* is the length of $L_{\text{ini}}^{\left(m-1\right)}$;
Step 3: optimize each shuffled TL in $L_{\text{ini}}^{\left(m\right)}$ by the *step2side* algorithm;
Step 4: terminate the algorithm if the increased *m* does not result in any improvement or if the current entanglement is zero. Otherwise, go to Step 2.

Just as with the *stepBothSides* algorithm, also *shufS2S* yields zero entanglement when applied to the Iris example visualized in Figure 1. However, the increase in computational cost with tree size is lower for *shufS2S*, and we will see later that on other datasets it tends to give lower entanglements. Due to their similarities, we will discuss the application and computational cost of the *shufS2S* algorithm together with the discussion on the algorithm introduced in the next section.

## 6 The shuffle & untangle algorithm

The example visualized in Figure 3 highlighted why *step2side* fails to untangle the drawn tanglegram. The discussion above also highlighted that *stepBothSides* improves the performance of *step2side* (as in Fig. 4), and that it may be extended opportunely to gain even more flexibility in searching for maximally untangled representations. Due to the computational cost of *stepBothSides*, we introduced the shuffle method in the previous section. In this section, we alter the previous method by replacing the *step2side* submethod by a new untangle method, and call this novel strategy Shuffle & Untangle (abbreviated later on with *ShUnTan*). Below we show that this scheme tends to give slightly better results than *shufS2S*, however at an increased computational cost.

### 6.1 The *ShUnTan* algorithm in pseudo-code

From a bird-eye viewpoint *ShUnTan* performs similar steps as in *shufS2S*, except for a new untangle scheme in Step 3. More precisely, *ShUnTan* implements the following operations, starting from an initial tanglegram $L_{0}=[Z_{l},Z_{r}]$:

Step 1: initiate *m* = 0 and $L_{\text{ini}}^{\left(0\right)}=\{L_{0}\}$;
Step 2: increase *m* by 1 and shuffle all TL(s) in $L_{\text{ini}}^{\left(m-1\right)}$, i.e. $L_{i}\in L_{\text{ini}}^{\left(m-1\right)}$, at the ${\left(n-m\right)}^{th}$ interior vertex by using the swap function $\mu_{n-m}$
  (6) $$L_{\text{ini}}^{\left(m\right)}=\overset{k}{\underset{i=1}{\cup }}\mu_{n-m}(L_{i}),\;L_{i}\in L_{\text{ini}}^{\left(m-1\right)},$$
  where *k* is the length of $L_{\text{ini}}^{\left(m-1\right)}$;
Step 3: optimize each shuffled TL in $L_{\text{ini}}^{\left(m\right)}$ by an approach will be explained in detail below to choose the best optimized layout;
Step 4: terminate the algorithm if the target layout is found or the increased *m* does not result in any improvement. Otherwise, go back to Step 2.

We note that Step 3 above builds a new set $L_{\text{unt}}^{\left(m\right)}$ by taking each element in $L_{\text{ini}}^{\left(m\right)}$ and applying to it two consecutive untangling operations. More precisely, assume *L* to be the element in $L_{\text{ini}}^{\left(m\right)}$ under consideration, and remember the definition of ξ(·) in (2). Then:

Substep 3.1: (symmetric optimization) Find the optimal layout L′ by optimizing the layout *L* as

(7a) $$L^{\left(1\right)}=\xi \left(\mu_{1}(L)\right)$$

(7b) $$L^{\left(2\right)}=\xi \left(\mu_{2}(L^{\left(1\right)})\right)$$

⋮

(7c) $$L\prime =L^{\left(n-m-1\right)}=\xi \left(\mu_{n-m-1}(L^{\left(n-m-2\right)})\right).$$

After (7c), assign L′ to *L* and repeat this sequence of equations until no further improvements can be made. Note that the operator *μ* is executed in the opposite direction of how it was used to find $L_{\text{ini}}^{\left(m\right)}$, starting from the leaves *μ*_1_ and moving towards the root. Also, the rotations *μ_i_* happen simultaneously at the same interior vertices for both the dendrograms. This is different from *step2side* and *stepBothSides*, where the former applies rotations to only one tree, while the later rotates both trees at all pairs of vertices;

Substep 3.2: (asymmetric optimization) build the new temporary set $L^{(m)\prime }$ starting from L′ computed in the substep above and operating as follows:

- for every object k=1,…,n (i.e. for each individual leaf node) find $\tau (k,Z_{l})$ and $\tau (k,Z_{r})$ (note that these indices may be different in the two dendrograms of L′);
- for each couple of such indexes $\tau (k,Z_{l})$ and $\tau (k,Z_{r})$ compute a new tanglegram through the asymmetric rotations function $\mu_{\tau (k,Z_{l}),\tau (k,Z_{r})}\left(L\prime \right)$;
- repeat the two steps above until the resulted layout is unchanged in two consecutive iterations, and then update $L^{(m)\prime }$ to be this final layout. The asymmetric rotation was introduced after observing that it could lead to further improvements;

Substep 3.3: add the best element in $L^{(m)\prime }$ to the set $L_{\text{unt}}^{\left(m\right)}$.

Just as with *stepBothSides* and *shufS2S*, also *shUnTan* yields zero entanglement when applied to the Iris example visualized in Figure 1. However, its computational cost is even higher than the one from *shufS2S*.

### 6.2 Some comments on the parameter *m* used in *shufS2S* and *ShUnTan*

The index *m* is likely the most relevant parameter among the ones characterizing algorithms embedding the ‘shuffle’ step, and it plays a key role in defining how large the search space is. In Step 2 of both *shufS2S* and *ShUnTan*, the starting value of *m* is 1, meaning that both algorithms first consider all the potential swappings at the root of the two dendrograms, getting thus $L_{\text{ini}}^{\left(1\right)}$ including four different layouts. By increasing *m* by 1 (and thus iterating Step 2), we take each of the four layouts in $L_{\text{ini}}^{\left(1\right)}$, considering all the potential swappings at the second last vertex of the two dendrograms, getting thus a $L_{\text{ini}}^{\left(2\right)}$ that includes 16 layouts, and so on. Note that this means shuffling starting from the outer sides of the tanglegram ($\mu_{n-1}$) and continuing towards its leafs. Briefly put, *m* indicates how far we shuffle the tanglegram from the root. For *ShUnTan*, moreover, *m* defines also how deep the algorithm should untangle the shuffled layouts starting from the first interior vertex. More precisely, *m* has the following simultaneous roles for every *L* in $L_{\text{ini}}^{\left(m\right)}$:

1. during the initialization *m* defines the number of interior vertices that will be shuffled in the following steps;
2. during the shuffling performed in Step 2, *m* affects the usage of the function *μ* [that in its turn shuffles the TL *L*_0_ at *m* interior vertices from the root to get a set of $4^{m}$ different TLs including the layout *L*_0_, see Equation (6)];
3. during the optimization process performed in Step 3, *m* reveals the amount of interior vertices (i.e. n−1−m) that are not examined in Step 2, and thus determines the number of times the Equations (7a)–(7c) are being computed;
4. *m* does however not impact the asymmetric optimization process, as this optimization step scans through all the potential labels independently of *m*.

We also note that increasing *m* reduces the number of times the operations in (7a)–(7c) are executed, but increases the number of permutations to be computed in Step 2. Importantly, it is possible to pre-set a limit to the computational costs by introducing a cut-off value $\bar{m}$ that terminates the execution of the algorithm when *m* increases too high. This is an additional possibility compared to *stepBothSides*, as both these algorithms suffer from a steep increase in computational time with the size of the trees. For the cases, we tested the algorithm converged before reaching high values of *m*, typically for *m* around 2 or 3. However, we note that some outliers with higher values of *m* occurred, with associated high computational cost. We note that it is immediate to force the algorithm to continue to higher values of *m* and check whether the overall results improve; however, it is outside the scope of this article investigating automatic strategies for determining optimal *m*-values from the data.

## 7 Experimental results

**Fig. 5.**
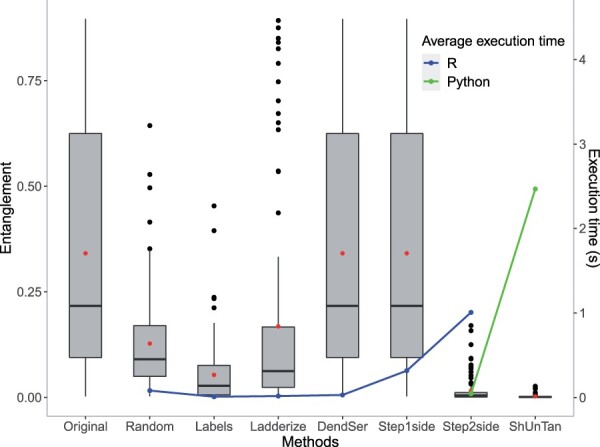
Comparison of the optimal entanglements and average execution times of the existing untangle methods in the dendextend *R* package. The dots inside the boxplots represent the average values of each group of optimal entanglements computed with each method. The experiment was carried on 100 random tanglegrams stemming from the Iris flower dataset (Fisher, 1936)

Figure 5 indicates *step2side* as the best (in terms of lowest reached entanglement levels) method in the dendextend *R* package, at the highest computational cost. As shown in the same plot, the computational cost for *shUnTan* is even higher for this example with 20 leaves. We also recall that applying *step2side* to the motivating example in Figure 1 led to the result shown in Figure 2, while applying *stepBothSides* led to Figure 4 with a drawable layout. As with *stepBothSides*, also *shufS2S* and *ShUnTan* reach the global optimum, a situation for which there is zero entanglement, i.e. a drawable layout.

We are thus interested in characterizing when our introduced methods yield a lower entanglement than *step2side*. To this aim, we performed an experimental comparison using Wisconsin Breast Cancer Database as a primary source of information (Wolberg and Mangasarian, 1990). More precisely, the experiment was performed on various trees sizes. For each of them (20, 40, 60 and so on), we then consider 40 different tanglegrams obtained implementing single and complete hierarchical clustering obtained using random samples from the Wisconsin Breast Cancer Database. We note that the actual source of data is irrelevant for the purpose of comparing *ShUnTan* against other untangle algorithms, since the unique object that is manipulated by these methods is actually the topology of the resulting dendrograms (in other words, we used the Breast Cancer Database as a proxy to generate 40 random initial tanglegrams for a range of different leaf sizes). We then compare the entanglement values and average execution time for *step2side*, *stepBothSides*, *shufS2S* and *ShUnTan*.

**Fig. 6.**
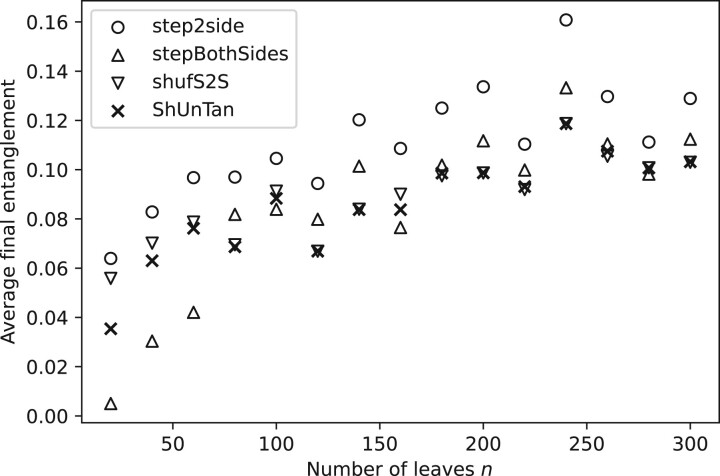
Average optimized entanglement of 40 random tanglegrams generated from the Wisconsin Breast Cancer Database (Wolberg and Mangasarian, 1990) as a function of the number of leaves (from 20 to 300)

Figure 6, that plots the average entanglement levels obtained for each untangle method for different dendrograms sizes, shows that the *step2side* method returns the highest entanglements amongst all studied algorithms. These entanglements are clearly lowered by other methods. In particularly, for small tree sizes, i.e. 20, 40 and 60 leaves, *stepBothSides* tends to improve the results of *step2side* most effectively. For larger trees, the algorithms embedding the shuffle step tend to give better results than *step2side* and *stepBothSides*. Between *shufS2S* and *ShUnTan*, the latter is markedly better than the former for small tree sizes, while such difference vanishes as the tree sizes enlarges.

A reasonable explanation for why the two algorithms embedding the shuffling step, i.e. *shufS2S* and *ShUnTan*, typically achieve a better final entanglement is that they use a more thorough strategy to optimize the entanglement value. As mentioned before, *step2side* only optimizes the original TL, while *shufS2S* and *ShUnTan* first generate a set of TLs from the original tanglegram by shuffling it at one or several interior vertices from the root, and then optimize every single TL in this set. Thus, *shufS2S* and *ShUnTan* also examine layouts that may be obtained by changes that actually lead to larger entanglements, and this enables them to exit local minima. *step2side* is not provided with this capability, and by considering successions of TLs whose entanglement is smaller and smaller it is more prone to local optima, as the search path cannot switch to layouts that might provide a path to more optimal layouts.

**Fig. 7.**
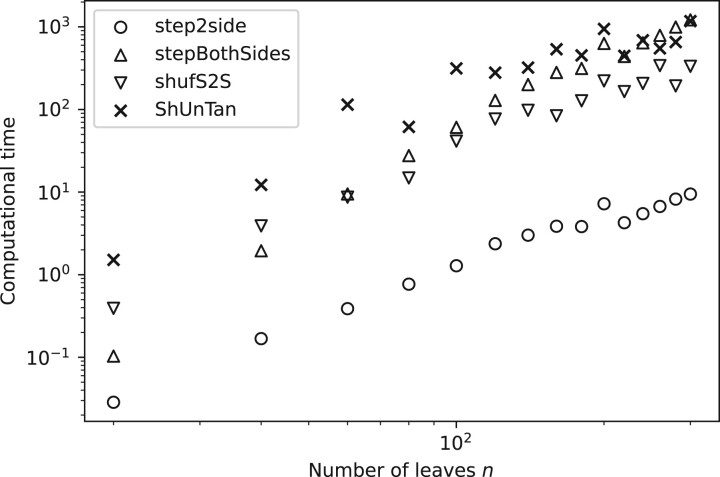
Empirical average computational time to obtain the optimized entanglement values, computed from the information plotted in Figure 6

Figure 7 shows the average computational time for the different algorithms and tree sizes, and highlights that all the introduced algorithms have higher computational cost than *step2side*. The figure moreover shows that the costs associated to the non-shuffling methods follow a power law trend—a phenomenon to be discussed below.

**Fig. 8.**
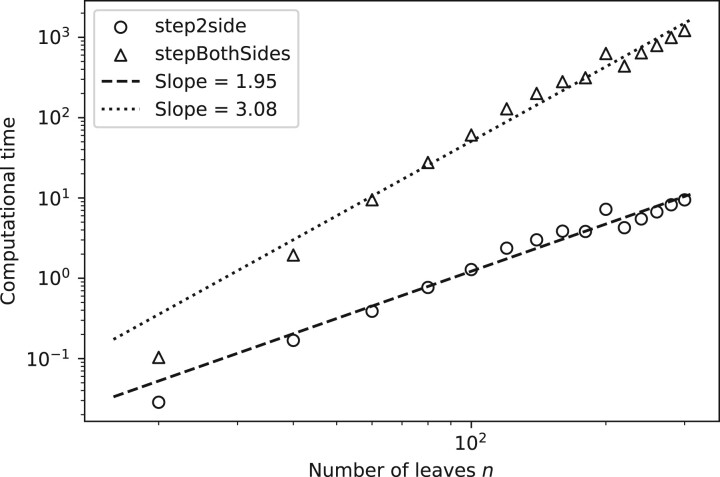
Average computational time for the *step2side* and *stepBothSides* methods, together with fitted slopes in log–log space

In terms of execution times, we first note that the average computational time for *stepBothSides* grows one order faster than *step2side*, i.e. as $O(n^{3.08})$, compared to $O(n^{1.95})$, as shown in Figure 8. Note that the slope values are dependent on the dataset, as we obtained slightly different slopes when we used the Iris dataset. This is in line with the number of test cases, being $O(n^{2})$ for *stepBothSides* versus O(n) for *step2side*.

**Fig. 9.**
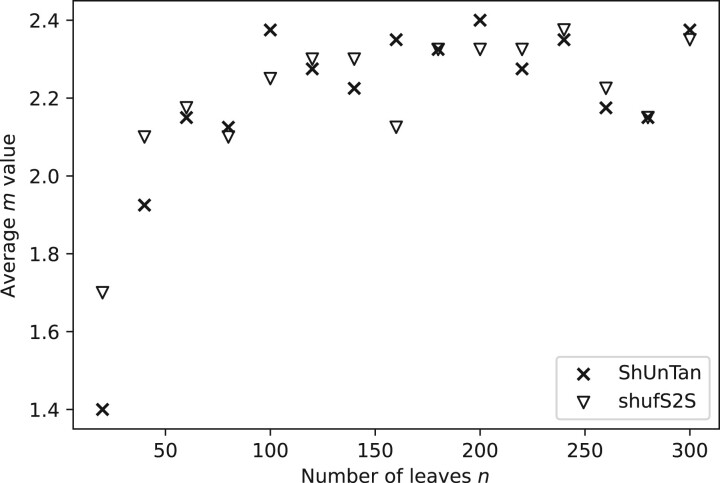
Average *m*-value versus tree size for the two shuffling algorithms

The computational cost of the two algorithms embedding the shuffling steps is not as easily analysable, as the computational time is heavily dependent on the depth given by the parameter *m*. Figure 9 plots the average *m*-value for the two shuffling algorithms for different tree sizes, and shows that after an initial rise, the average *m*-value seems to stabilize.

**Fig. 10.**
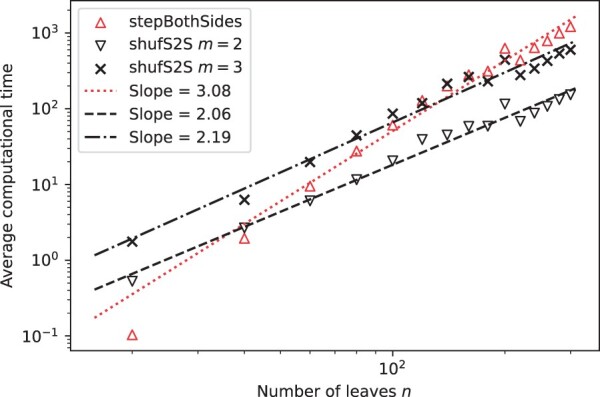
Computational time of *shufS2S* with different values of *m*, together with the times associated to *stepBothSides*

Plotting then the computational time for subsets with different *m*-values, it is possible to note that such computational times follow specific power laws. Figure 10, that plots the computational time for *shufS2S* for two different *m*-values, shows indeed that both slopes are lower than the slope for *stepBothSides*, which indicates that *shufS2S* will overtake *stepBothSides* in computational efficiency if the average *m*-value is stable. Indeed, the *shufS2S* algorithm has typically a lower computational cost than *stepBothSides* from around 60 leaves.

Moreover, the *ShUnTan* algorithm associates to slopes of 2.00 and 2.02 for *m* = 2 and 3, respectively. Such slopes are lower than the ones associated to *shufS2S*, and this indicates that the former typically requires lower computational times for larger tree sizes. However, cross-overs were not observed up to a tree size of 300. Finally, while the computational cost of *ShUnTan* is higher than *shufS2S* for our data, it has lower computational cost than *stepBothSides* for the largest tree sizes in our dataset.

## 8 Conclusions

This study introduced a few alternative algorithms to address the TL problem, and compared them against existing untangle methods available in *R* (in particular *step2side*, that was selected as a benchmark as it was the best performing algorithm among the existing ones in *R* for our test sets).

We highlighted how, despite being selected as benchmark, *step2side* may perform poorly and stop in local minima with high entanglement values also on simple tanglegrams. We thus identified how to enhance it by introducing opportune steps that enable exiting combinations associated with local minima. Summarizing, the critical point, we identified was the need for introducing methods swapping vertices in the two trees simultaneously.

Such swapping steps were then implemented by our proposed algorithms, *stepBothSides*, *shufS2S* and *ShUnTan*, all of which were shown capable of untangling crossings that *step2side* fails to eliminate. Unfortunately, the lower entanglements obtained by these new methods come at a high computational cost relative to the *step2side* method.

More precisely, our findings suggest that the *ShUnTan* algorithm produces the lowest entanglement values among all these algorithms. The *stepBothSides* method instead tends to perform better than *ShUnTan* for networks up to around 60 leaves, and gradually worse as the size increases—but still better than *step2side*. The computational requirements of *stepBothSides* reflect the same structure: it is smaller than the shuffling methods up to around 60 leaves, after which it is overtaken by the *shufS2S* algorithm first, and later by the *ShUnTan* algorithm too. The novel proposed algorithms are thus deemed to be useful in situations where users are most interested in obtaining a better final result than having fast computations. Moreover, if time requirements are not an issue, then *ShUnTan* is preferrable for larger tree sizes.

Future works include the characterization of the trade-offs introduced by the parameters that define the novel algorithms, and find implementation strategies that may reduce their computational costs without leading to adverse effects on their final results.
